# Beyond Binary Cutoffs: An Explainable Machine Learning Framework for Individualized Diagnostic Reasoning in Suspected Urolithiasis

**DOI:** 10.3390/diagnostics16091313

**Published:** 2026-04-27

**Authors:** Kyungman Cha, Sang Hoon Oh, Jaekwang Shin, Jee Yong Lim

**Affiliations:** 1 Department of Emergency Medicine, Suwon St. Vincent Hospital, The Catholic University of Korea, Suwon 16247, Republic of Korea; drchaa@catholic.ac.kr; 2 Department of Emergency Medicine, Seoul St. Mary’s Hospital, The Catholic University of Korea, Seoul 06591, Republic of Korea; 3 Department of Sports and Technology, Seokyeong University, Seoul 02713, Republic of Korea; 4 International Healthcare Center, Seoul St. Mary’s Hospital, The Catholic University of Korea, Seoul 06591, Republic of Korea

**Keywords:** urolithiasis, explainable artificial intelligence, SHAP, clinical prediction rule, diagnostic uncertainty, Shannon entropy, gradient boosting

## Abstract

**Background**: Emergency department evaluation of suspected urolithiasis increasingly relies on non-contrast CT, yet not all patients require imaging. Existing clinical prediction rules help stratify stone probability, but by converting continuous measurements into fixed binary indicators, they offer little insight into why a particular patient is at risk or how much uncertainty remains after each testing stage—questions that bear directly on individualized diagnostic decisions. **Methods**: We retrospectively analyzed 1000 ED patients with suspected urolithiasis who underwent non-contrast CT (stone prevalence 85.0%). A gradient boosting classifier was trained on 17 continuous clinical and laboratory features and compared against binary-thresholded counterparts and an established scoring system; the 17-feature model achieved AUC 0.771 (95% CI 0.726–0.813) versus 0.723 (95% CI 0.675–0.771) for the reference score on this cohort (DeLong *p* = 0.001). Individual predictions were explained using an interventional Shapley value approach, and a Shannon entropy-based framework was applied to quantify the marginal diagnostic contribution of each sequential testing stage. **Results**: Held-out permutation importance identified red blood cell count on microscopy, age, pain duration, and prior stone history as the most influential predictors. Several features showed non-linear contributions that diverged from conventional binary thresholds: creatinine effect crossed zero near 0.90 mg/dL and pain duration peaked between 2 and 5 h. C-reactive protein, absent from existing scoring systems, emerged as a meaningful negative predictor. Sequential entropy analysis showed that dipstick urinalysis provided the largest marginal information gain among non-history stages (6.1% of prior entropy), while physical examination contributed 2.3%. A prevalence sensitivity analysis projected that the framework’s threshold behavior would differ substantially in lower-prevalence populations, underscoring that the cohort-specific cut-points are not portable decision rules. We therefore position the framework as a reasoning aid that complements clinical judgment and imaging, not as a stand-alone triage tool. **Conclusions**: Explainable machine learning can address questions that aggregate discrimination metrics cannot: which features drive risk for a given patient, how those effects behave across the continuous measurement range, and how much diagnostic uncertainty each testing stage resolves. The Shapley-based explanations and entropy framework developed here offer a structured approach to individualized diagnostic reasoning in the ED evaluation of suspected urolithiasis, functioning as an interpretive adjunct to, rather than a replacement for, existing clinical tools and CT imaging.

## 1. Introduction

Urolithiasis remains one of the most common reasons for emergency department visits involving acute flank pain, and its prevalence continues to rise worldwide [1,2]. While non-contrast computed tomography (CT) is the reference standard for diagnosis, concerns about radiation exposure, cost, and scanner availability have motivated the development of clinical prediction rules that might identify patients at sufficiently high or low probability of harboring a stone to justify modifying the imaging strategy [3,4,5].

Several such scoring systems have been proposed, typically assigning points to a small number of binary variables derived from history, physical examination, urinalysis, and blood tests [6,7]. These instruments have demonstrated reasonable discriminative performance across different ED populations and offer the practical advantage of bedside applicability without computational tools. Both instruments convert continuous or ordinal measurements into binary indicators using predefined cutoffs, a step that simplifies bedside use but inevitably discards information.

The consequences of dichotomization in clinical prediction have been discussed extensively in the statistical literature [8]. A creatinine value of 0.91 mg/dL and one of 0.93 mg/dL are treated identically when separated by a threshold of 0.92, even though their true association with stone probability may differ only marginally. Similarly, pain duration is often treated as a simple binary (<8 h vs. ≥8 h), despite the possibility that the relationship between symptom duration and stone probability is non-monotone. Such information loss is the price paid for simplicity, and whether the trade-off is worthwhile depends on how much predictive value is actually being sacrificed.

Machine learning (ML) models offer an alternative approach that can accommodate continuous variables and capture non-linear or interactive effects without requiring a priori cutoff selection [9,10]. However, the clinical utility of ML in this context is often questioned on two grounds. First, the discriminative improvement over well-designed scoring systems tends to be modest in moderate-sized single-center datasets. Second, the “black box” nature of many ML algorithms undermines the clinical transparency that scoring systems inherently provide [11,12]. Recent work has increasingly addressed this tension by pairing tree-based classifiers with Shapley-based explanation: van Doorn et al., for instance, developed a multicenter explainable ML model for rapid risk stratification of ED patients using routine laboratory data and SHAP-based interpretation [13], and Moreno-Sánchez et al. applied XGBoost with SHAP to ED patient flow prediction on the MIMIC-IV dataset [14].

Explainable artificial intelligence (XAI) techniques, particularly those grounded in Shapley value theory, have been increasingly adopted to address the interpretability concern [15,16]. By decomposing a model’s prediction into feature-level contributions for each individual patient, these approaches can answer clinically meaningful questions: not just “what is this patient’s probability of having a stone?” but also “which specific findings are driving that estimate, and in which direction?” The broader role of AI as a diagnostic support tool in clinically ambiguous presentations has been reviewed in other domains, including thyroid cytopathology [17], and the urolithiasis-specific ML literature has expanded substantially in recent years, with work by Nedbal et al. on ureteroscopic outcomes [18] and Kim et al. on deep-learning-based stone detection in ED CT images [19]. These efforts have focused predominantly on imaging-based or interventional outcomes rather than on the pre-imaging, bedside diagnostic-reasoning problem that motivates our study. To our knowledge, this perspective has not been applied to urolithiasis diagnosis in the ED setting.

Beyond explainability, a question that existing prediction rules leave largely unanswered is how much additional diagnostic information each successive test contributes for a given patient. In routine ED practice, the diagnostic workup follows a roughly sequential pattern: history, physical examination, dipstick urinalysis, microscopy, and blood tests. Yet few studies have quantified the marginal information gain at each step or identified the point at which further testing no longer meaningfully reduces diagnostic uncertainty.

In this study, we pursued three related aims. First, we compared the discriminative performance of a gradient boosting classifier trained on continuous features against binary-thresholded counterparts and an established scoring system. Second, we used an interventional Shapley value approach to characterize how individual features contribute to predictions, with particular attention to non-linear effects that diverge from conventional dichotomization thresholds. Third, we developed a Shannon entropy-based sequential framework to quantify the marginal diagnostic value of each testing stage and to identify patients whose diagnostic uncertainty remains irreducible despite all available bedside information. Our intent is not to propose a replacement for existing clinical tools but rather to illustrate how explainable ML might complement them by offering a more granular, patient-level understanding of the diagnostic process.

## 2. Materials and Methods

### 2.1. Study Design and Population

**Figure 1:**
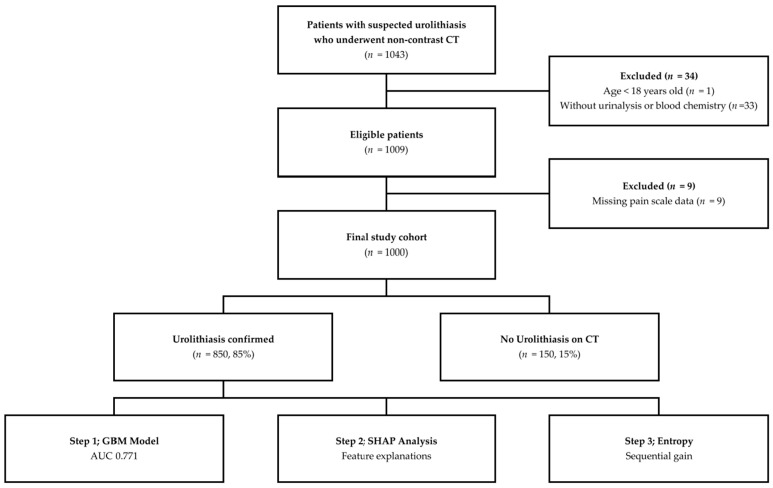
Study flow diagram. A total of 1043 patients with suspected urolithiasis who underwent non-contrast CT were identified. After exclusion of 34 patients (1 aged <18, 33 without urinalysis or blood chemistry) and 9 with missing pain scale data, 1000 patients constituted the final study cohort (850 [85.0%] with confirmed urolithiasis). The analytic pipeline comprised three sequential steps: gradient boosting model development, Shapley-based explainability analysis, and entropy-based sequential information gain assessment.

This was a retrospective, single-center study conducted at the ED of Seoul St. Mary’s Hospital, a tertiary referral center affiliated with The Catholic University of Korea. We reviewed the records of consecutive adult patients (≥18 years) who presented with acute flank or abdominal pain suggestive of urolithiasis and underwent non-contrast CT between January 2014 and December 2015. A total of 1043 patient records were identified. After applying exclusion criteria (age < 18 years, *n* = 1; missing urinalysis or blood chemistry data, *n* = 33), 1009 patients remained eligible. Of these, 9 patients had missing values for the pain scale variable and were excluded, yielding a final cohort of 1000 patients (Figure 1). Complete-case analysis was used because the proportion of missing data was small (0.9%) and the missing variable was a primary predictor rather than a covariate amenable to imputation without introducing systematic bias. The reference standard for urolithiasis was the presence of a ureteral or renal calculus on non-contrast CT as interpreted by board-certified radiologists. Patients without a directly visualized stone but with ipsilateral acute obstructive signs on CT—including hydronephrosis, perinephric stranding, or ureteral dilatation—were also classified as having urolithiasis, as these findings are recognized indicators of urinary tract obstruction and are clinically indistinguishable from cases in which the stone has passed prior to imaging. The study was approved by the Institutional Review Board (IRB) of Seoul St. Mary’s Hospital (KC26RASI0131) and was conducted in accordance with the Declaration of Helsinki. Informed consent was waived by the IRB due to the retrospective nature of the study.

### 2.2. Predictor Variables

Seventeen clinical and laboratory variables were selected based on their availability in routine ED evaluation and their inclusion in prior scoring systems. These comprised five history and demographic features (age, sex, prior stone history, pain duration in hours, pain scale), four physical examination findings (nausea, vomiting, costovertebral angle tenderness, body temperature), three dipstick urinalysis results (leukocyte esterase, specific gravity, occult blood), three microscopy findings (red blood cell count, white blood cell count, crystalluria), and two blood test results (serum creatinine, C-reactive protein). All continuous variables were retained in their original scale without dichotomization.

For comparison, two additional reference models were constructed using the same nine clinical variables in binary form, mirroring the structure of an established scoring system [7]: one applying predefined dichotomization cutoffs (9-feature binary model) and one retaining the original continuous values (9-feature continuous model). This allowed us to isolate the contribution of feature continuity from that of feature selection.

### 2.3. Model Development

We trained a gradient boosting classifier (scikit-learn GradientBoostingClassifier, version 1.3) on the 17 features. Gradient boosting constructs an ensemble of shallow decision trees in a stage-wise additive fashion, with each successive tree fitted to the pseudo-residuals of the current ensemble. Unlike single decision trees, this approach can approximate complex non-linear and interaction effects while remaining more interpretable than deep neural networks. Hyperparameters were selected to balance model expressiveness against overfitting risk in a moderately sized, single-center dataset. Specifically, we used 200 boosting iterations with a conservative learning rate of 0.05 to allow gradual, stable convergence; a maximum tree depth of 3 to limit individual tree complexity; a minimum leaf size of 30 samples to prevent fitting to sparse subgroups; and stochastic subsampling of 80% of training instances and square-root feature selection per split to introduce regularizing variance across trees. No explicit grid search was performed; instead, hyperparameters were fixed a priori based on general gradient boosting literature, prioritizing stability over marginal performance gains.

Model performance was evaluated using five-fold stratified cross-validation. Predicted probabilities from the held-out folds were aggregated to compute the area under the receiver operating characteristic curve (AUC-ROC) with 95% confidence intervals derived from 1000 bootstrap samples [20,21]. Calibration was assessed visually using quantile-based calibration plots and numerically using the Brier score [20]. The two 9-feature reference models described in Section 2.2 were trained under identical cross-validation settings, enabling direct comparison of the effect of feature continuity and feature selection on discriminative performance.

Permutation importance was computed on the held-out test fold of each of the five cross-validation splits, using 30 permutation repeats per fold and a fixed random seed, and reported as the mean and standard deviation across folds. Computing the metric on held-out rather than in-sample data ensures that the reported importance reflects each feature’s contribution to out-of-sample discrimination rather than in-sample fit.

As a transparent baseline for comparison, we additionally trained a logistic regression classifier on the same 17 features under identical five-fold stratified cross-validation, with all continuous features standardized prior to fitting (zero mean, unit variance). This allowed us to quantify the incremental discriminative contribution of the gradient boosting model relative to a simple linear model fitted to the same inputs. Differences in out-of-fold AUC between the two models were assessed using a paired bootstrap procedure (2000 resamples) to obtain a confidence interval and *p*-value for the AUC difference.

### 2.4. Explainability Analysis

To explain individual predictions, we employed an interventional approach to computing Shapley-based feature contributions [22]. Shapley values, originating from cooperative game theory, provide a theoretically principled decomposition of a model’s output into additive contributions from each input feature, satisfying properties of efficiency (contributions sum to the prediction), symmetry, and null player consistency [15,16,23]. For each patient and each feature, the contribution was estimated by marginalizing over a reference distribution of 100 randomly sampled background observations: the feature of interest was replaced with background values while all other features were held at their observed values, and the mean change in predicted probability was recorded as that feature’s contribution. Summing contributions across all features reconstructs the difference between the individual’s predicted probability and the population mean. This interventional framing—sometimes termed the “do-calculus” SHAP—is preferred over the conditional expectation approach when features are correlated, because it avoids attributing variance to a feature through its statistical associations with other inputs, yielding contributions that are less driven by correlations among features and may better approximate each feature’s marginal contribution under the interventional SHAP framework [24].

The 100-observation background sample was drawn by simple random sampling without replacement from the 1000-patient cohort, with a fixed random seed for reproducibility. A sensitivity check with alternative background sample sizes (50, 100, and 200) yielded essentially the same global feature ranking, confirming that the Shapley estimates were not sensitive to the specific background size within this range. Because the interventional formulation introduces some approximation relative to the exact tree-based computation, we quantified the gap between the sum of Shapley contributions and the model’s predicted probability on each patient. The average absolute reconstruction error across the cohort was 0.069, which we treat as the expected cost of the interventional formulation relative to the exact TreeSHAP algorithm. The practical consequence for interpretation—namely, that feature-value zero-crossings should be read as transition regions rather than as precise cut-points—is addressed in the Limitations section.

To identify non-linear feature effects that differ from conventional binary thresholds, we examined dependence plots relating each continuous feature’s value to its Shapley contribution across the cohort. Rolling mean trends were overlaid to smooth sample-level noise and to identify inflection points—values at which a feature’s contribution crosses zero or changes direction—that may diverge from the fixed cutoffs used in existing scoring systems.

Four representative patients were selected to illustrate the clinical relevance of individual-level explanations: (A) a classic stone presentation with high predicted probability, (B) a correctly low-probability non-stone case, (C) a diagnostically uncertain case with conflicting feature contributions, and (D) a stone mimicker with high probability but no confirmed stone on CT.

### 2.5. Sequential Information Gain Framework

To quantify the marginal diagnostic value of each testing stage, we adapted a Shannon entropy-based information gain framework [20]. For each patient at each stage, diagnostic uncertainty was expressed as binary entropy H(p) = −p·log_2_(p) − (1 − p)·log_2_(1 − p), where p is the model-predicted probability of urolithiasis given the features available at that stage. Entropy is maximized at 1.0 bit when p = 0.5 (complete uncertainty) and approaches 0 as the prediction converges toward 0 or 1. The prior entropy H_0_, computed from the observed cohort prevalence (85.0%), served as the baseline against which all subsequent entropy reductions were referenced (H_0_ = 0.610 bits). This prevalence-anchored baseline ensures that marginal gains are interpreted relative to what a clinician would know before any testing, rather than against an uninformative 50% prior.

Five sequential testing stages were defined to mirror the typical ED workflow: Stage 1 (history and demographics), Stage 2 (adding physical examination), Stage 3 (adding dipstick urinalysis), Stage 4 (adding microscopy), and Stage 5 (adding blood tests). At each stage, a separate gradient boosting model was trained using only the features available up to that point. The marginal information gain of each stage was computed as the reduction in mean population-level entropy relative to the prior stage.

Each stage-specific model was trained with the same hyperparameters as the full 17-feature model, differing only in the feature subset available. Predicted probabilities at each stage were obtained from the held-out fold of the same five-fold stratified cross-validation used throughout the study, and all entropy calculations were performed on these held-out predictions. Because the stage-specific models are trained independently, the stage-to-stage changes in an individual patient’s predicted probability do not necessarily decompose into additive contributions from the newly added features alone; they also incorporate any re-weighting of earlier features induced by the expanded feature set. The interpretive consequences of this property are addressed in the Limitations section.

At the patient level, we defined “confident classification” as a predicted probability exceeding 90% (high-probability group) or falling below 20% (low-probability group) at any stage. Patients reaching these thresholds at an earlier stage were considered resolved and excluded from subsequent stages. The proportion of patients who did not reach either threshold after all five stages was taken as an estimate of the population for whom clinical and laboratory evaluation alone could not resolve diagnostic uncertainty.

### 2.6. Statistical Analysis

Continuous variables are presented as mean ± standard deviation or median (interquartile range) as appropriate. Categorical variables are presented as counts and percentages. AUC comparisons between the gradient boosting model and the established reference score were performed using DeLong’s test. All analyses were conducted in Python 3.10 using scikit-learn 1.3, NumPy 1.24, and pandas 2.0. A two-sided *p*-value < 0.05 was considered statistically significant.

## 3. Results

### 3.1. Study Population

The final cohort comprised 1000 patients (660 [66.0%] male; mean age 47.5 ± 13.8 years). Urolithiasis was confirmed in 850 patients (85.0%). Patients with confirmed urolithiasis were more likely to be male (67.9% vs. 55.3%), to have a prior stone history (31.5% vs. 12.7%), and to have higher serum creatinine (1.0 ± 0.3 vs. 0.9 ± 0.3 mg/dL). Median pain duration was shorter in the stone group (2.0 vs. 3.0 h). Detailed baseline characteristics are presented in Table 1.

### 3.2. Model Performance

**Figure 2:**
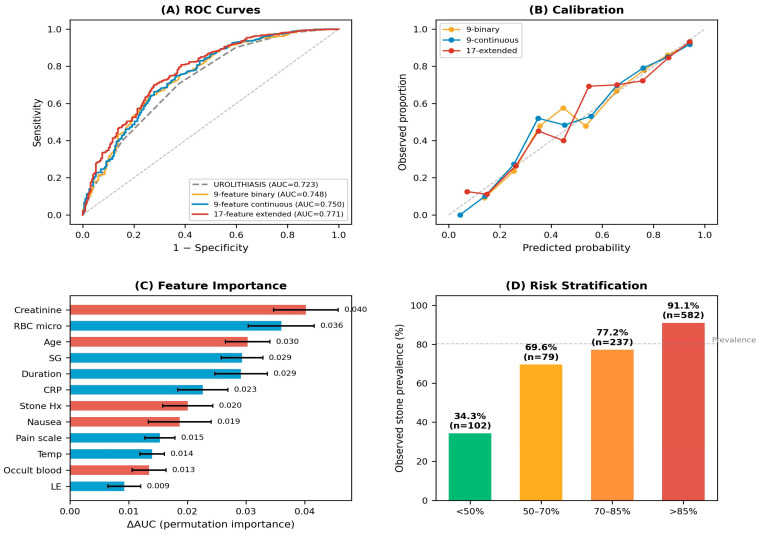
Model performance and risk stratification. (**A**) Receiver operating characteristic curves for the established reference scoring system (AUC 0.723) and three gradient boosting models: 9-feature binary (AUC 0.748), 9-feature continuous (AUC 0.750), and 17-feature extended (AUC 0.771, 95% CI 0.726–0.813) models. The 17-feature model achieved a statistically higher AUC than the reference score by DeLong’s test (*p* = 0.001), though the absolute difference was modest. (**B**) Calibration plots showing observed stone proportion versus predicted probability for each ML model. (**C**) Held-out permutation importance of the top 12 features in the 17-feature extended model, computed on the held-out test fold of each of five cross-validation splits (30 permutation repeats per fold); error bars represent the standard deviation of the per-fold means. (**D**) Observed stone prevalence across four risk strata defined by the extended model’s predicted probability.

On five-fold cross-validation, the 17-feature gradient boosting model achieved an AUC of 0.771 (95% CI, 0.726–0.813; Brier score 0.102), compared with 0.750 for the 9-feature continuous model (Brier 0.104) and 0.748 for the 9-feature binary model (Brier 0.105). When the established reference scoring system was applied to this cohort, it achieved an AUC of 0.723 (95% CI, 0.675–0.771); the difference between the 17-feature model and the reference score was statistically significant by DeLong’s test (*p* = 0.001), though the extended model did not demonstrate a clinically meaningful improvement in discrimination that would justify its complexity over a simpler scoring approach. Calibration curves indicated reasonable agreement between predicted probabilities and observed stone rates across all three ML models, though all tended to slightly underestimate risk in the highest probability decile (Figure 2A,B).

A logistic regression classifier trained on the same 17 features under identical cross-validation settings achieved an out-of-fold AUC of 0.760 (95% CI, 0.713–0.803; Brier score 0.107). The paired bootstrap comparison of gradient boosting against logistic regression yielded a mean ΔAUC of +0.012 (95% CI, −0.027 to +0.050; bootstrap *p* = 0.54); that is, the advantage of gradient boosting over a simple linear baseline was not statistically significant in this cohort. This finding reinforces the interpretation that, at the level of aggregate discrimination, the value added by ML over a transparent baseline is modest; the substantive contribution of the framework lies in the interpretive analyses that follow.

Held-out permutation importance, averaged across the five cross-validation folds, identified serum creatinine as the most informative feature (mean ΔAUC 0.040), followed by red blood cell count on microscopy (0.036), age (0.030), specific gravity (0.029), pain duration (0.029), C-reactive protein (0.023), prior stone history (0.020), and nausea (0.019). Leukocyte esterase and dipstick occult blood showed lower but non-zero contributions. Full per-fold values are provided in Supplementary Table S1. This ranking highlights the central role of biochemical and microscopic urinary signals in model discrimination; notably, CRP—absent from all published urolithiasis scores—ranked sixth and remains among the top contributors (Figure 2C).

When patients were stratified by predicted probability, the model effectively separated risk groups. Patients classified as low-risk (<50%) had a stone prevalence of 34.3% (*n* = 102), those at moderate risk (50–70%) had a prevalence of 69.6% (*n* = 79), those at high risk (70–85%) had a prevalence of 77.2% (*n* = 237), and those at very high risk (>85%) had a prevalence of 91.1% (*n* = 582) (Figure 2D).

### 3.3. Shapley-Based Feature Explanations

#### 3.3.1. Global Feature Effects

**Figure 3:**
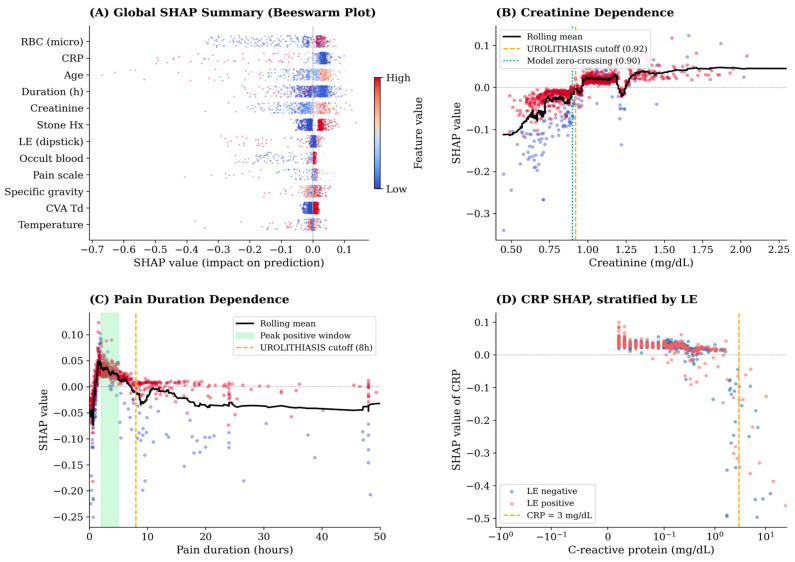
Shapley-based feature effects. (**A**) Global feature importance: mean absolute SHAP value across all 1000 patients. (**B**) Shapley dependence plot for serum creatinine, with rolling mean trend line (black) and conventional binary threshold of 0.92 mg/dL (dashed orange); each point is a patient, colored by stone status. (**C**) Shapley dependence plot for pain duration, showing peak positive contribution in the 2–5 h window (shaded green) rather than at the conventional 8 h cutoff (dashed orange). (**D**) SHAP contribution of CRP plotted against CRP value, stratified by leukocyte esterase status (blue: LE negative; red: LE positive). The dashed orange line marks CRP = 3 mg/dL.

The global Shapley analysis (Figure 3A) identified RBC microscopy and serum creatinine as the two features with the largest mean absolute contributions to individual predictions (mean |SHAP| 0.044 and 0.037, respectively). In the beeswarm plot, high creatinine values consistently shifted predictions toward urolithiasis, while low values had a strong opposing effect. RBC microscopy showed a graduated, dose-dependent pattern: counts of zero carried the strongest negative contribution, progressively attenuating through intermediate categories, with counts ≥100 providing only a modest positive contribution.

#### 3.3.2. Non-Linear Effects and Cutoff Discrepancies

Examination of Shapley dependence plots (Figure 3B,C) revealed several non-linear relationships that differed from the fixed binary thresholds used in conventional scoring systems.

Creatinine: The Shapley contribution crossed zero at approximately 0.90 mg/dL, slightly below the conventional threshold of 0.92 mg/dL. Above 1.2 mg/dL, the positive contribution plateaued, suggesting diminishing marginal returns at very high values. This pattern implies that creatinine values between 0.90 and 0.92 already carry positive predictive information that the binary cutoff discards.

Pain duration: Rather than a simple dichotomy at 8 h, the model identified a non-monotone relationship. Shapley contributions were most positive (favoring stone) in the 2–5 h window (stone prevalence 91.2%), crossing zero around 5 h and becoming progressively negative for durations exceeding 16 h (stone prevalence 67.2%). This suggests that the relevant clinical signal is not merely “short” versus “long” duration but rather a specific time window during which stone-related colic is most characteristic.

C-reactive protein: Although CRP is not included in any existing urolithiasis scoring system, the model assigned it substantial negative importance at elevated levels. Patients with CRP values above 3 mg/dL had a stone prevalence of only 45.5% (mean SHAP −0.209), compared with 86.1% at CRP levels below 0.5 mg/dL. Elevated CRP likely reflects competing inflammatory or infectious etiologies, making it a clinically meaningful low-probability signal that current scores miss entirely. Detailed stratified SHAP contributions and stone prevalence by feature value intervals are presented in Table 2.

#### 3.3.3. Distinguishing CRP from Leukocyte Esterase

Because CRP and urinary leukocyte esterase might be expected to co-vary in an inflammatory context, we examined how the model distinguishes their contributions. In our cohort the two features were essentially uncorrelated (Spearman ρ = 0.031, *p* = 0.33), and the per-patient Shapley contributions for the two features were likewise uncorrelated (Spearman ρ = 0.027, *p* = 0.39), indicating that the gradient boosting model treats them as distinct sources of information rather than as statistical substitutes.

Stratifying the cohort into four subgroups defined by CRP (≤3 vs. >3 mg/dL) and leukocyte esterase (negative vs. positive) clarified how the two contributions combine. In the CRP > 3 mg/dL group, the mean Shapley contribution of CRP was essentially the same whether leukocyte esterase was positive (mean SHAP_CRP_ = −0.247, *n* = 11) or negative (mean SHAP_CRP_ = −0.232, *n* = 10)—that is, the model’s negative attribution to CRP is not mediated by concurrent leukocyturia. When both markers were present and pointing the same direction, the model additionally attributed a separate negative contribution to leukocyte esterase (mean SHAP_LE_ = −0.091); when CRP was elevated but leukocyte esterase was negative, the leukocyte esterase contribution was effectively zero (mean SHAP_LE_ = −0.012). Figure 3D shows the joint distribution and subgroup means. Clinically, these patterns are consistent with CRP and leukocyte esterase capturing distinct aspects of an inflammatory picture: systemic CRP elevation is treated by the model as intrinsically suggestive of an alternative diagnosis, while leukocyte esterase contributes incremental evidence only when actively positive.

#### 3.3.4. Individual Patient Explanations

**Figure 4:**
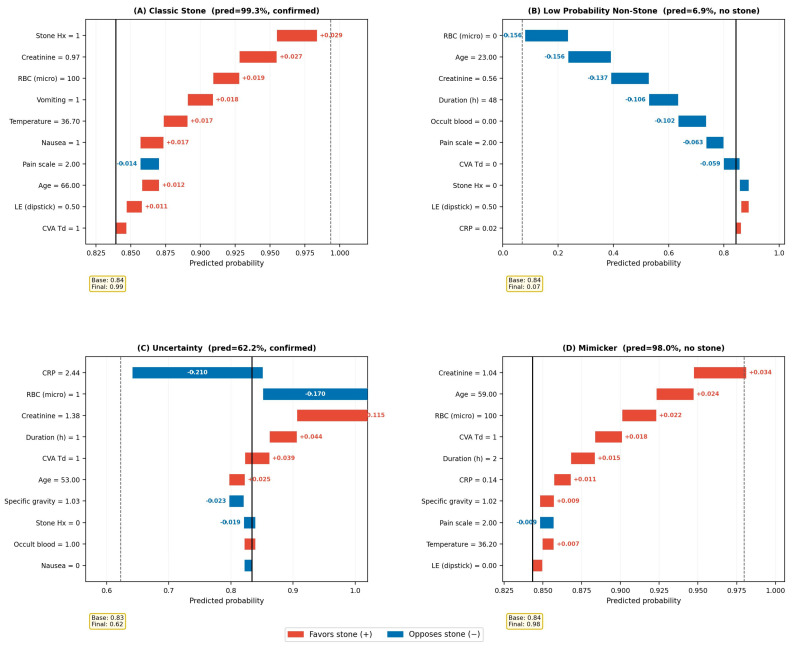
Individual patient explanations (waterfall plots). Four representative cases illustrating feature-level contributions: (**A**) classic stone presentation with concordant positive features; (**B**) low-probability non-stone case with concordant negative features; (**C**) diagnostic uncertainty driven by conflicting contributions from elevated creatinine (favoring stone) and elevated CRP and leukocyte esterase (opposing); (**D**) stone mimicker with uniformly stone-like features but no stone on CT. Red bars indicate features favoring urolithiasis; blue bars indicate features opposing it.

The clinical utility of individual-level Shapley explanations is illustrated through four cases selected to represent distinct explanatory patterns (Figure 4). These cases are intended as illustrative examples and do not constitute a claim of representativeness.

Case A (classic stone, predicted 99.4%, confirmed) presented with a typical profile: prior stone history (SHAP +0.006), nausea (SHAP +0.004), age of 66 years (SHAP +0.003), and creatinine of 0.97 mg/dL (SHAP +0.003). All features pointed in the same direction, and the model expressed high confidence with an identifiable rationale.

Case B (low predicted probability, 5.3%, no stone confirmed) was a 23-year-old woman with low creatinine (0.56 mg/dL, SHAP −0.152), negative occult blood (SHAP −0.081), and zero RBC on microscopy (SHAP −0.076). Young age (SHAP −0.052) and prolonged duration of 48 h (SHAP −0.048) further contributed. The convergence of strong negative signals drove the prediction well below the 20% low-probability threshold.

Case C (uncertainty, predicted 53.5%, confirmed stone) demonstrates where explainability adds the most clinical value. This 67-year-old man had elevated creatinine (1.47 mg/dL, SHAP +0.088) and age-related positive contributions (+0.081) that favored stone diagnosis. However, markedly elevated CRP (3.57 mg/dL, SHAP −0.254) and leukocyte esterase (2+, SHAP −0.236) pushed the prediction strongly in the opposite direction. The waterfall plot makes explicit what a numerical score cannot: this patient’s intermediate probability is not a result of “not enough evidence” but of strong evidence pointing in conflicting directions. The appropriate response is not more bedside testing but rather definitive imaging to resolve the tension between stone-suggestive and infection-suggestive findings.

Case D (mimicker, predicted 94.6%, no stone on CT) had stone-like features throughout, including elevated creatinine, maximal hematuria, and high occult blood, yet harbored no stone. The model’s high confidence was understandable given the available data, but the case illustrates a fundamental limitation: when all measurable features point toward a stone, no amount of ML sophistication can detect a stone mimicker without imaging.

### 3.4. Sequential Information Gain

**Figure 5:**
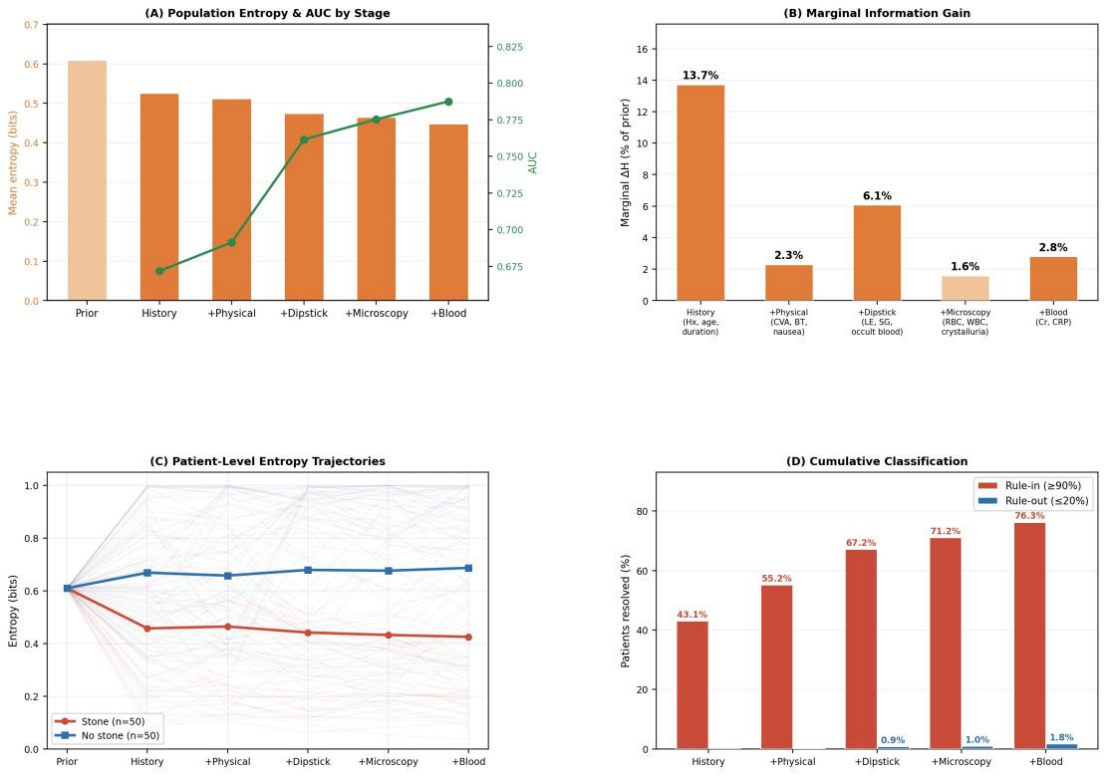
Sequential information gain analysis. (**A**) Mean population-level entropy (bars) and AUC (green line) at each sequential testing stage. (**B**) Marginal entropy reduction contributed by each new testing stage. (**C**) Patient-level entropy trajectories for stone (red) and non-stone (blue) patients. (**D**) Cumulative proportion of patients reaching confident classification at each stage.

The prior entropy based on the population stone prevalence of 85.0% was 0.610 bits. Adding the five testing stages progressively reduced mean entropy to 0.444 bits, representing a cumulative 27.2% reduction in diagnostic uncertainty (Table 3, Figure 5A).

The marginal contribution of each stage was not uniform (Table 3, Figure 5B). History and demographics (Stage 1) provided the largest single reduction (13.7% of prior entropy), driven primarily by age, stone history, and pain duration. Physical examination (Stage 2) added 2.3%, consistent with its known limited discriminative value in this setting. Dipstick urinalysis (Stage 3) contributed the second-largest increment (6.1%), chiefly through occult blood and leukocyte esterase. Microscopy (Stage 4) added 1.6%, and blood tests (Stage 5) added 2.8%. Notably, the AUC progression showed a similar pattern: the largest stepwise jump occurred at Stage 3 (AUC 0.691 → 0.766, Δ = +0.075), while adding microscopy to dipstick results improved AUC by only 0.012.

At the individual patient level, entropy trajectories diverged markedly between confirmed stone and non-stone patients (Figure 5C). For stone patients, mean entropy decreased steadily from 0.610 to approximately 0.45 bits across the five stages, reflecting progressive accumulation of confirmatory evidence. For non-stone patients, however, mean entropy remained flat or increased slightly, indicating that the high prior probability (85.0%) made “ruling out” a stone far more difficult than ruling it in. This asymmetry highlights a structural challenge: in a high-prevalence population, even substantial negative evidence may be insufficient to overcome the prior.

**Figure 6:**
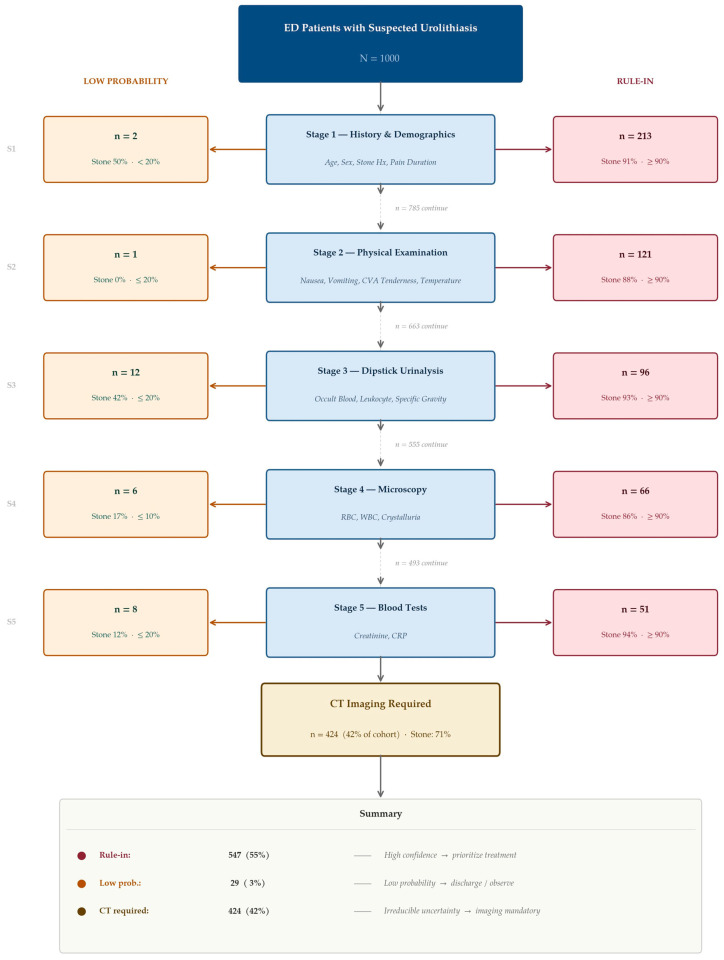
Illustrative XAI-guided sequential diagnostic framework. Schematic representation of the proposed sequential diagnostic strategy, showing patient flow through five testing stages with high-probability (≥90%) and low-probability (≤20%) exit thresholds at each stage.

Using thresholds of 90% (high-probability) and 20% (low-probability), 43.1% of patients reached a model-based classification threshold after Stage 1 (history and demographics) alone, predominantly via the high-probability pathway. Successive stages progressively expanded this proportion to 55.2% after physical examination, 67.2% after dipstick urinalysis, 71.2% after microscopy, and 78.1% after blood tests. At the final stage, 76.3% fell into the high-probability group and 1.8% into the low-probability group (Figure 5D). Applying these final classifications to the original cohort yielded 547 patients (55%) in the rule-in group, 29 (3%) in the low-probability group, and 424 (42%) who did not reach either threshold after all five stages and for whom imaging remained necessary to resolve residual uncertainty (Figure 6). Within the final low-probability group (predicted probability ≤ 0.20), observed stone prevalence varied across the sequential stages and ranged from 12% to 50% (Figure 6), reflecting the difficulty of excluding a stone in a cohort with an 85% prior; as discussed in Section 4, this is in large part a structural consequence of the high baseline prevalence rather than a property of the model itself.

At a more permissive threshold (85%/30%), 58.2% of patients reached the high-probability boundary and 3.6% the low-probability boundary, leaving 38.2% without a confident model-based classification. The stone prevalence among patients remaining unresolved after threshold-based stratification was 69.6%, indicating that the framework preferentially selects a diagnostically challenging subpopulation for imaging (Figure 6).

## 4. Discussion

In this study, we applied an explainable machine learning framework to the emergency department diagnosis of urolithiasis and found that its primary value lies not in superior aggregate discrimination but in three complementary contributions: revealing non-linear feature effects that diverge from conventional cutoffs, providing transparent individual-level explanations for clinical predictions [15], and quantifying the marginal information gain of sequential diagnostic testing [25,26].

The finding that the ML model did not demonstrate a clinically meaningful improvement over a well-designed scoring system is itself informative. Several methodological factors may explain this. The dataset of 1000 patients, while adequate for model training, may be insufficient for a gradient boosting classifier to fully exploit the added complexity of 17 continuous features relative to a well-calibrated binary score; simulations suggest that tree-based ML models require substantially larger training sets to consistently outperform simpler rule-based approaches in binary clinical outcomes [27,28]. Additionally, some of the continuous variables included—such as specific gravity and urine WBC—may carry relatively high measurement noise in routine ED settings, attenuating the expected benefit of retaining them on their original scale. These observations suggest that existing scoring systems already capture the predominant predictive signal in this population, and that the comparative advantage of ML in this context lies in interpretability and individualization rather than aggregate discrimination [27].

Two additional comparisons support this interpretation. First, a logistic regression model trained on the same 17 continuous features achieved an out-of-fold AUC of 0.760 (95% CI, 0.713–0.803), and the paired bootstrap comparison against gradient boosting yielded ΔAUC = +0.012 (95% CI, −0.027 to +0.050; *p* = 0.54), which is not statistically significant. That a transparent linear baseline matches the gradient boosting model on aggregate discrimination reinforces the view that the incremental value of this framework in the present cohort lies elsewhere. Second, the 9-feature continuous model attained an AUC of 0.750, modestly below the 17-feature model’s 0.771 but well within the bootstrap confidence intervals of either model. The eight additional variables in the 17-feature set offered only marginal discriminative gain on their own, consistent with the observation that their signal-to-noise ratio in routine ED practice is modest relative to the core features. We present the 17-feature model because its richer feature set supports the Shapley-based non-linear and individual-level analyses that follow, not because it outperforms simpler alternatives on aggregate metrics.

The non-linear effects identified through Shapley analysis have several practical implications. The creatinine zero-crossing near 0.90 mg/dL, rather than 0.92, suggests that a modestly lower threshold might capture additional predictive information without meaningfully increasing false positives. The peak predictive window for pain duration at 2–5 h, rather than the blanket < 8 h cutoff, aligns with the typical timeline of ureteral peristaltic colic and may reflect the clinical reality that very short presentations (<1 h) are less discriminating because stone and non-stone etiologies both present acutely, while very long presentations (>16 h) may indicate a complication or alternative diagnosis [29]. Most notably, the strong negative contribution of CRP at levels above 3 mg/dL, which reduced stone prevalence from 86.1% to 45.5%, highlights a clinically recognizable but formally unaccounted-for signal. In our cohort, among the 11 non-stone patients with CRP above 3 mg/dL, the most common alternative findings included urinary tract infection or cystitis (*n* = 3), pelvic inflammatory disease (*n* = 1), pyelonephritis (*n* = 1), and benign prostatic hyperplasia with suspected prostatitis or cystitis (*n* = 2). This distribution is consistent with the interpretation that markedly elevated CRP should shift clinical suspicion toward infectious or inflammatory mimickers rather than uncomplicated stone disease, echoing prior observations that infectious etiologies are among the most common alternative diagnoses in patients initially suspected of urolithiasis [30].

The individual patient explanations (Figure 4) illustrate a distinct mode of clinical reasoning that numerical scores do not support. When a patient falls into an intermediate risk stratum on a conventional scoring system, the clinician knows the probability range but little else. The Shapley waterfall plot, by contrast, reveals whether the intermediate probability results from uniformly mild evidence or from strong conflicting signals, a distinction with direct implications for next steps. Case C exemplifies this: the opposing contributions of elevated creatinine and elevated CRP make explicit the clinical tension between stone-suggestive and infection-suggestive findings, flagging the patient for definitive imaging rather than additional bedside tests.

The sequential information gain analysis provides a quantitative basis for a question that clinicians navigate intuitively but rarely formalize: how much does each additional test contribute for this patient? Our finding that dipstick urinalysis provided the largest marginal gain among non-history stages, while microscopy added comparatively little incremental value, is consistent with the general clinical intuition that dipstick hematuria is one of the most informative bedside findings in suspected urolithiasis [31]. The minimal incremental gain from microscopy over dipstick (ΔAUC = 0.008, entropy reduction 1.3%) raises a clinically meaningful question for resource-constrained ED settings: whether routine urine microscopy adds sufficient diagnostic information to justify its time and cost in all patients with suspected urolithiasis, or whether it could be safely reserved for those with equivocal dipstick findings or intermediate predicted probability. This preliminary observation merits prospective investigation.

The asymmetric entropy trajectories between stone and non-stone patients deserve emphasis. In a population with 85% stone prevalence, the prior already strongly favors a stone, making confirmation relatively straightforward while exclusion requires overcoming this prior with consistently negative evidence. This structural asymmetry has direct implications for the 20% low-probability threshold: because the pre-test probability is so high, even a model prediction below 20% still carries a non-trivial stone rate (observed stone prevalence in OOF-predicted <20% group: 20.0%, *n* = 10), and cannot be used as a clinical rule-out. The 20% boundary was chosen to represent a theoretically meaningful probability reduction from the 85% prior, not to define a clinically safe discharge threshold. Projected predictive values across a range of alternative baseline prevalences are presented in Supplementary Table S3; external application of this framework requires recalibration to the local prevalence of the target population. We therefore frame this framework not as a triage rule but as a reasoning aid: a way of making explicit which features are driving a prediction, how much uncertainty remains after each testing stage, and where the points of diagnostic tension lie for a given patient. Whether the 90% and 20% thresholds or any other decision boundaries are clinically actionable is a separate question that depends on the local prevalence, the consequences of misclassification, and prospective validation, none of which this study can answer.

To illustrate how the framework’s behavior would change in populations with more typical ED stone prevalence, we projected the positive and negative predictive values of the 0.90 and 0.20 thresholds across a range of baseline prevalences using the observed sensitivities and specificities in our held-out predictions. At the cohort’s observed prevalence of 85%, the positive predictive value of a predicted probability ≥ 0.90 was 94.1% and the negative predictive value of a predicted probability ≤ 0.20 was 86.7%. At a hypothetical 40% prevalence (closer to an unselected ED flank-pain population), the positive predictive value would fall to 65.3% while the negative predictive value would rise to 98.2%; at 30% prevalence, the corresponding values would be 54.8% and 98.8% (Supplementary Table S3). This asymmetry is informative: the difficulty of exclusion observed in our cohort is in large part a structural artifact of the extreme prior probability, and in lower-prevalence populations the same threshold structure would be expected to behave very differently. We emphasize that these projections are analytical extrapolations rather than direct evidence; prospective validation in an unselected population is required before any threshold can be proposed for clinical use, and the small number of patients in the ≤0.20 bin of our cohort limits the precision of the underlying specificity estimate.

This study has several limitations that should be acknowledged. First, the gradient boosting model, despite achieving a statistically higher AUC by DeLong’s test (0.771 vs. 0.723, *p* = 0.001), did not demonstrate a clinically meaningful improvement in aggregate discrimination over the reference scoring system, suggesting that the added complexity of ML is unlikely to be justified on discrimination grounds alone in this setting. Second, it is a single-center retrospective analysis, and the results require external validation before any clinical application can be considered. The high stone prevalence (85.0%) reflects the selection of patients who underwent non-contrast CT at a tertiary referral center; this is substantially higher than estimates from unselected ED flank pain cohorts, where stone prevalence typically ranges from 30 to 50%. At lower baseline prevalence, the positive predictive value of the high-probability threshold would diminish markedly, and the baseline entropy would be closer to its theoretical maximum of 1.0 bit, offering greater potential for information gain. The prior entropy of 0.610 bits observed in this study therefore reflects a high-prevalence population in which meaningful entropy reduction is already structurally limited. Furthermore, nine patients with missing pain scale data were excluded via complete-case analysis; a comparison of these patients against the included cohort (Supplementary Table S2) showed that they differed systematically on several characteristics, most notably having a lower observed stone prevalence (44.4% vs. 85.0%, *p* = 0.006) and an older age distribution, a pattern consistent with atypical presentations in which pain was not the dominant chief complaint. Given the small proportion affected (0.9%), the impact on the trained model is likely modest, but we note this difference explicitly rather than treating the missingness as uninformative. Third, the Shapley values were computed using an interventional marginalization approach, introducing an average reconstruction error of 0.069 between the sum of Shapley contributions and the model’s predicted probability. This has a practical consequence for regions in which a feature’s Shapley contribution is small in absolute magnitude—which is precisely the case near the zero-crossings we identified. For borderline values such as creatinine around 0.90 mg/dL or pain duration in the 2–5 h window, the precise location of the inflection point should therefore be read as an indicative transition region rather than a precise cut-point, and formal threshold determination for clinical use would require exact TreeSHAP and bootstrap confidence intervals on the crossing location. Fourth, we did not account for potential temporal trends in practice patterns or changes in CT utilization over the study period. Fifth, the stage-specific models in the sequential framework were trained independently at each stage, which means that adding a new testing result at a later stage can implicitly re-weight the contributions of earlier features. The marginal information gain we attribute to each stage therefore reflects the total entropy reduction associated with that stage, which includes both the direct contribution of the newly added features and any re-weighting of earlier features that those additions induce. A fully decomposed attribution—separating the independent contribution of each stage from cross-stage interaction effects—would require a different formulation, such as conditional Shapley values applied to the full model, and should be pursued in future work. Finally, as Case D illustrates, any prediction model is bounded by the information content of its inputs; when all measurable features resemble stone, no statistical method can distinguish a true stone from a perfect mimicker without imaging.

## 5. Conclusions

Explainable machine learning, applied to the emergency department diagnosis of urolithiasis, provides a complementary lens to conventional scoring systems. Its primary contributions lie not in overall discriminative performance but in three specific dimensions: uncovering non-linear feature effects that binary cutoffs obscure, offering transparent patient-level explanations that distinguish between different sources of diagnostic uncertainty, and quantifying the marginal information gain of each sequential diagnostic step. In this high-prevalence ED population, the framework exposed non-linear feature effects and individual-level sources of diagnostic uncertainty that aggregate scoring systems cannot reveal; it is best understood as a reasoning aid that complements clinical judgment and imaging, not as a replacement for them. The model’s cohort-specific probability thresholds are not portable decision rules: their behavior depends strongly on local prevalence, and their clinical utility requires prospective validation in populations that differ from ours in both case mix and baseline stone probability. Whether these insights translate into actionable changes in clinical workflow will require prospective evaluation and, ultimately, integration with the clinical judgment of the emergency physician who knows the patient.

## Figures and Tables

**Table 1:** Baseline characteristics of the study population stratified by urolithiasis status on CT.

Variable	Stone (*n* = 850)	No Stone (*n* = 150)	Test	*p*-Value
Demographics				
Age, years	48.1 ± 13.2	44.4 ± 16.6	t	**0.002**
Male sex, *n* (%)	577 (67.9)	83 (55.3)	χ^2^	**0.009**
Prior stone history, *n* (%)	268 (31.5)	19 (12.7)	χ^2^	**<0.001**
Pain duration, h, median (IQR)	2.2 (1.1–5.7)	3.8 (1.0–17.1)	U	**0.006**
Pain scale (0–10)	5.0 ± 2.2	4.6 ± 2.3	U	**0.030**
Symptoms and Physical Examination				
Nausea, *n* (%)	172 (20.2)	16 (10.7)	χ^2^	**<0.001**
Vomiting, *n* (%)	112 (13.2)	8 (5.3)	χ^2^	**0.001**
CVA tenderness, *n* (%)	517 (60.8)	72 (48.0)	χ^2^	0.004
Body temperature, °C	36.5 ± 0.4	36.6 ± 0.6	U	**0.003**
Dipstick Urinalysis				
Occult blood ≥1+, *n* (%)	791 (93.1)	104 (69.3)	χ^2^	**<0.001**
Leukocyte esterase ≥1+, *n* (%)	92 (10.8)	30 (20.0)	χ^2^	0.002
Specific gravity	1.020 ± 0.008	1.019 ± 0.008	U	0.393
Urine Microscopy				
RBC (/HPF), median (IQR)	50 (20–100)	20 (1–100)	U	**<0.001**
WBC (/HPF), median (IQR)	0 (0–1)	0 (0–1)	U	0.171
Crystalluria, *n* (%)	76 (8.9)	3 (2.0)	χ^2^	**0.006**
Blood Tests				
Creatinine, mg/dL	1.0 ± 0.3	0.9 ± 0.3	U	**<0.001**
CRP, mg/dL, median (IQR)	0.08 (0.04–0.18)	0.10 (0.03–0.35)	U	0.034

Data are presented as mean ± SD, median (IQR), or *n* (%). t, Student’s *t*-test; χ^2^, chi-squared test; U, Mann–Whitney U test. Boldface *p*-values indicate statistical significance (*p* < 0.05). CVA, costovertebral angle; RBC, red blood cell; WBC, white blood cell; HPF, high-power field; CRP, C-reactive protein; LE, leukocyte esterase; SG, specific gravity.

**Table 2:** Non-linear feature effects: stratified Shapley contributions and observed stone prevalence by feature value intervals for creatinine, pain duration, and CRP.

Feature/Interval	*n*	Stone %	Mean SHAP ± SD	Conventional Cutoff	Interpretation
Creatinine (mg/dL)					
<0.70	109	71.9	−0.096 ± 0.069		Below both thresholds
0.70–0.87	207	80.4	−0.033 ± 0.048	<0.92 → 0	Model negative, score also 0
0.87–0.92	88	80.5	−0.013 ± 0.034	<0.92 → 0	Model borderline, score also 0
0.92–1.20	440	90.9	+0.044 ± 0.036	≥0.92 → 1	Both positive
>1.20	156	85.6	+0.036 ± 0.057	≥0.92 → 1	Plateau; diminishing returns
Pain Duration (hours)					
<1	163	83.1	−0.009 ± 0.032		Minimal signal in acute window
1–2	264	89.7	+0.001 ± 0.025	<8 h → 1	Borderline; approaching peak
2–5	268	91.2	+0.014 ± 0.021	<8 h → 1	Peak positive window
5–16	182	83.8	−0.003 ± 0.042	<8 h or ≥8 h	Cross-zero; mixed signal
>16	123	67.2	−0.066 ± 0.069	≥8 h → 0	Strong negative; alt. diagnosis
CRP (mg/dL)					
<0.5	899	86.1	+0.003 ± 0.012	Not included	Low CRP favors stone
0.5–3.0	80	79.0	−0.064 ± 0.107	Not included	Moderate negative signal
>3.0	21	45.5	−0.209 ± 0.101	Not included	Strong low-probability signal

SHAP, Shapley additive explanation values (interventional); LE, leukocyte esterase; SG, specific gravity. Positive SHAP values favor urolithiasis; negative values oppose it. The reference cutoff column indicates the binary threshold applied in conventional scoring systems for each feature.

**Table 3:** Sequential information gain analysis across five diagnostic stages.

Diagnostic Stage	*n* Feat	AUC	Mean H (bits)	Marginal ΔH (%)	Cumul. ΔH (%)	Rule-in *n* (Stone %)	Low-Prob. *n* (Stone %)	Key Variables Added
Prior (prevalence)	—	—	0.610	—	—	—	—	—
Stage 1: History	5	0.666	0.521	14.6	14.6	454 (90.1)	1 (0.0)	Hx stone, pain duration, age
Stage 2: +Physical	9	0.691	0.509	2.0	16.6	569 (89.6)	1 (100.0)	CVA Td, nausea, vomiting
Stage 3: +Dipstick	12	0.766	0.469	6.6	23.1	692 (90.6)	9 (22.2)	Occult blood, LE, SG
Stage 4: +Microscopy	15	0.778	0.461	1.3	24.4	721 (90.7)	11 (27.3)	RBC, WBC, crystalluria
Stage 5: +Blood	17	0.793	0.444	2.8	27.2	762 (90.4)	19 (31.6)	Creatinine, CRP

*n* feat, cumulative number of features; H, Shannon entropy (bits); ΔH, entropy reduction as percentage of prior entropy (0.610 bits); AUC, area under the receiver operating characteristic curve; SG, specific gravity; LE, leukocyte esterase; BT, body temperature; Hx, history; CVA, costovertebral angle; Td, tenderness.

## Data Availability

The datasets generated and/or analyzed during the current study are not publicly available due to patient privacy and institutional restrictions. De-identified data may be made available from the corresponding author upon reasonable request and subject to Institutional Review Board approval and institutional data-sharing policies.

## Supplementary Materials

The following supporting information can be downloaded at https://www.mdpi.com/article/10.3390/diagnostics16091313/s1, Table S1. Held-out permutation importance of the top 12 features across five-fold cross-validation. Table S2. Comparison of baseline characteristics between included (*n* = 1000) and excluded (*n* = 9) patients. Table S3. Projected positive and negative predictive values of the 0.90 and 0.20 probability thresholds at alternative baseline stone prevalences.

## Abbreviations

The following abbreviations are used in this manuscript:

ED	emergency department
CT	computed tomography
ML	machine learning
XAI	explainable artificial intelligence
SHAP	SHapley Additive exPlanations
AUC	Area Under the (Receiver Operating Characteristic) Curve
ROC	Receiver Operating Characteristic
CRP	C-reactive protein
CVA	costovertebral angle
RBC	red blood cell
WBC	white blood cell
HPF	high-power field
IQR	interquartile range
