# Supplementary material for: Beyond Binary Cutoffs: An Explainable Machine Learning Framework for Individualized Diagnostic Reasoning in Suspected Urolithiasis
Source: Diagnostics (Basel). 2026 Apr 27;16(9):1313. doi: 10.3390/diagnostics16091313 (PMC13163005; doi:10.3390/diagnostics16091313)
Supplement: Supplementary file 1 [file diagnostics-16-01313-s001.zip › diagnostics-4223029-supplementary.pdf]

Supplementary Materials

Title: Beyond Binary Cutoffs: An Explainable Machine Learning Framework for Emergency Department Urolithiasis Diagnosis

Supplementary Table S1. Held-out permutation importance

Permutation importance of the top 12 features in the 17-feature gradient boosting model, computed on the held-out test fold of each of five stratified cross-validation splits (30 permutation repeats per fold). Values are mean ΔAUC and standard deviation across folds. These values are visualised in Figure 2C of the main manuscript.

**Feature**	**Mean ΔAUC**	**SD**
Serum creatinine	0.04	0.005
RBC microscopy	0.036	0.005
Age	0.03	0.004
Specific gravity	0.029	0.004
Pain duration	0.029	0.004
C-reactive protein	0.023	0.005
Prior stone history	0.02	0.003
Nausea	0.019	0.003
Pain scale	0.015	0.003
Temperature	0.014	0.002
Occult blood (dipstick)	0.013	0.002
Leukocyte esterase	0.009	0.002

Supplementary Table S2. Comparison of included and excluded patients

Comparison of baseline characteristics between the 1,000 patients included in the analysis and the 9 patients excluded due to missing pain scale data. Values are median (interquartile range) for continuous variables and n/N (%) for categorical variables. p-values are from Mann–Whitney U tests for continuous variables and Fisher's exact test or chi-squared test for categorical variables.

**Characteristic**	**Included (*n*=1000)**	**Excluded (*n*=9)**	***p*-value**
Age (years)	48.0 (38.0–57.0)	59.0 (54.0–71.0)	0.049
Pain duration (hours)	2.0 (1.0–6.0)	7.0 (3.0–20.0)	0.185
Body temperature (°C)	36.5 (36.1–36.7)	36.5 (36.2–36.9)	0.743
Serum creatinine (mg/dL)	1.0 (0.8–1.1)	1.0 (0.9–1.1)	0.535
C-reactive protein (mg/dL)	0.1 (0.0–0.2)	0.2 (0.1–0.9)	0.044
Male sex	660/1000 (66.0%)	9/9 (100.0%)	0.033
Prior stone history	287/1000 (28.7%)	1/9 (11.1%)	0.459
Nausea	188/1000 (18.8%)	0/9 (0.0%)	0.223
Vomiting	120/1000 (12.0%)	0/9 (0.0%)	0.61
CVA tenderness	589/1000 (58.9%)	2/9 (22.2%)	0.038
LE positive (≥1+)	122/1000 (12.2%)	0/9 (0.0%)	0.61
Urolithiasis on CT	850/1000 (85.0%)	4/9 (44.4%)	0.006

The excluded patients differed from the included cohort in several respects, most notably a lower observed stone prevalence (44.4% vs. 85.0%, p = 0.006), an older age distribution (median 59 vs. 48 years, p = 0.049), and lower rates of costovertebral angle tenderness (22.2% vs. 58.9%, p = 0.038). This pattern is consistent with atypical presentations in which pain was not the dominant chief complaint and in which a structured pain scale was therefore not documented.

Supplementary Table S3. Projected predictive values at alternative baseline prevalences

Projected positive and negative predictive values of the 0.90 and 0.20 probability thresholds under alternative hypothetical baseline stone prevalences. Projections use Bayesian reweighting of the observed sensitivity and specificity of each threshold in our held-out predictions (Se for ≥0.90 = 0.740, Sp = 0.707; Se for ≤0.20 = 0.989, Sp = 0.120).

Baseline stone prevalence	PPV of predicted probability ≥0.90	NPV of predicted probability ≤0.20
85%	93.5%	66.7%
70%	85.5%	82.9%
60%	79.1%	88.3%
50%	71.6%	91.9%
40%	62.7%	94.4%
30%	52.0%	96.4%
20%	38.7%	97.8%

These projections are analytical extrapolations based on the sensitivities and specificities observed in our cohort, and should not be interpreted as direct evidence of performance in low-prevalence populations. The small number of patients in the ≤0.20 bin of the present cohort (n = 27) also limits the precision of the specificity estimate for the low-probability threshold. Prospective validation in an unselected ED cohort is required before any threshold can be proposed for clinical use.
